# Supplementary material for: Coupling between oxygen redox and cation migration explains unusual electrochemistry in lithium-rich layered oxides
Source: Nat Commun. 2017 Dec 12;8:2091. doi: 10.1038/s41467-017-02041-x (PMC5727078; doi:10.1038/s41467-017-02041-x)
Supplement: Supplementary file 1 — Supplementary Information [file 41467_2017_2041_MOESM1_ESM.pdf]

Supplementary Figures

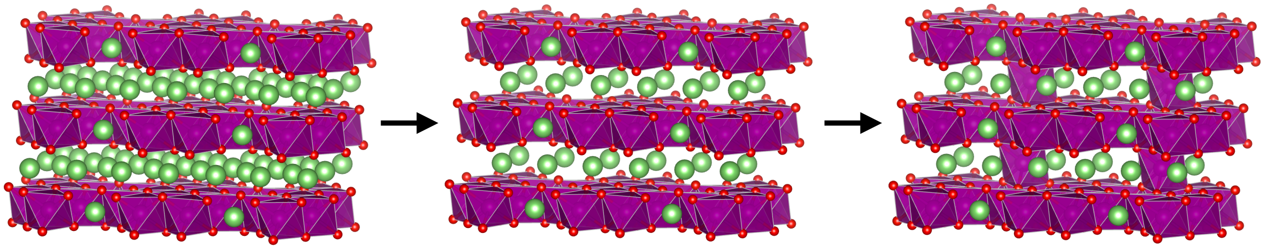

Supplementary Figure 1. TM Migration Schematic. Schematic showing TM migration into vacant Li sites in the interlayer space during delithiation. Green spheres represent Li, red spheres represent O, and purple spheres represent TMs. The purple polyhedra are TMO_6 octahedra.

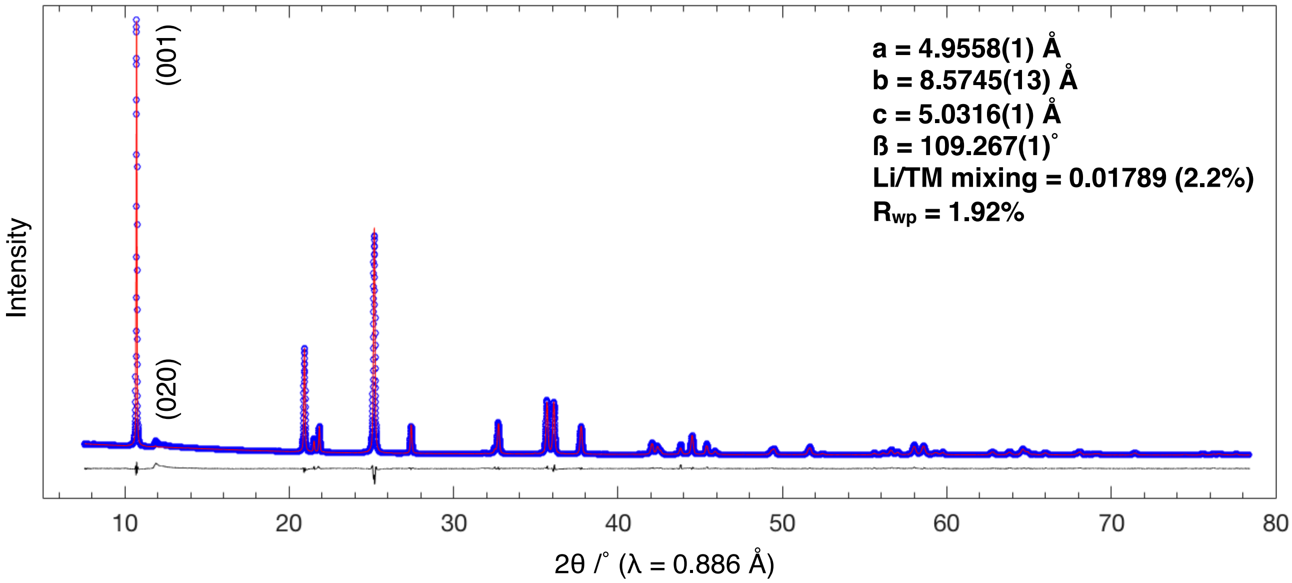

Supplementary Figure 2. Pristine Material Rietveld Refinement. Rietveld refinement of the XRD pattern of a pellet of the pristine LMR-NMC powder. All the peaks could be indexed to the monoclinic *C2/m* space group. The inter-layer spacing and fraction of TMs in the Li layer are consistent with previous results.[1-3] The region of the (020) superstructure peaks was not fit as these peaks are strongly affected by stacking faults[4] that are not accounted for in this refinement.

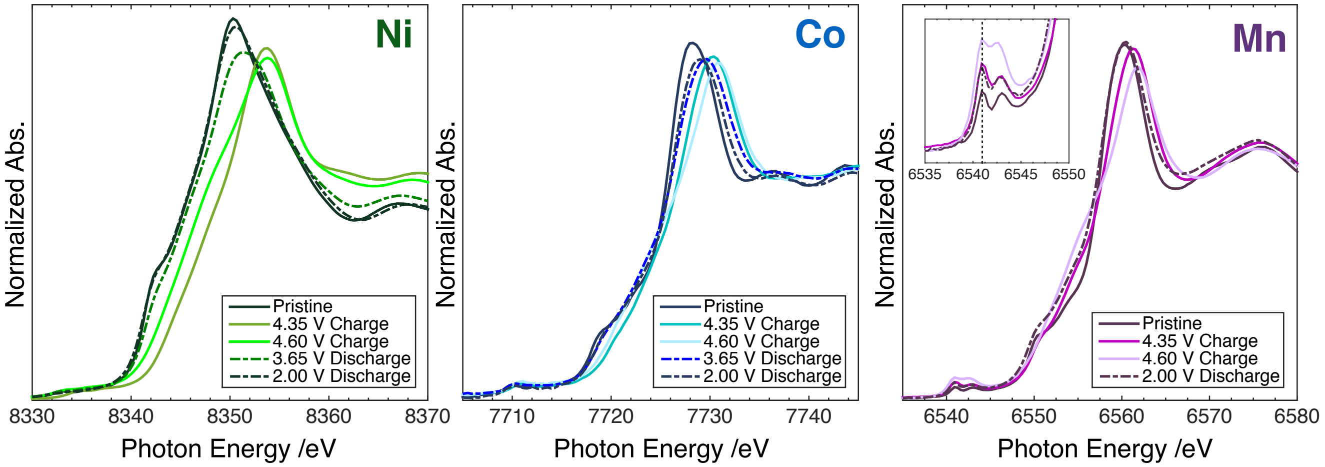

Supplementary Figure 3. TM K edge XAS throughout the First Cycle. Ni shows the largest oxidation below the plateau (< 4.35 V). The changes at the Ni and Mn edges during the plateau are consistent with literature reports[5] and EXAFS data (Supplementary Fig. 10) that show a significant change in the local coordination environment, likely due to migration of these TMs into sites in the Li layer. The large increase in the Mn pre-edge intensity is also consistent with a significant structural change during the plateau.[6] On discharge, consistent with the L_3 edge XAS taken from STXM (Figure 2b, main text), the Ni and Co K edges show only a small degree of reduction below 3.65 V during discharge, indicating that most of this capacity is hysteretic O redox. Consistent with the minimal change in the average Mn L_3 edge spectrum after the first cycle (Figure 2e, main text), the Mn pre-edge feature shows no shift, indicating that, when averaged throughout the entire material, Mn exhibits minimal overall redox activity during the first cycle.[3,6,7] The small shift of the Mn edge itself to lower energy at 2.00 V is likely due to the altered local structure as a result of TM migration, evident in the increased TM_{Li} fraction obtained from XRD (Figure 1c, main text), the reduced EXAFS first shell scattering intensity (Supplementary Fig. 10), and the increased Mn pre-edge intensity after the first cycle.

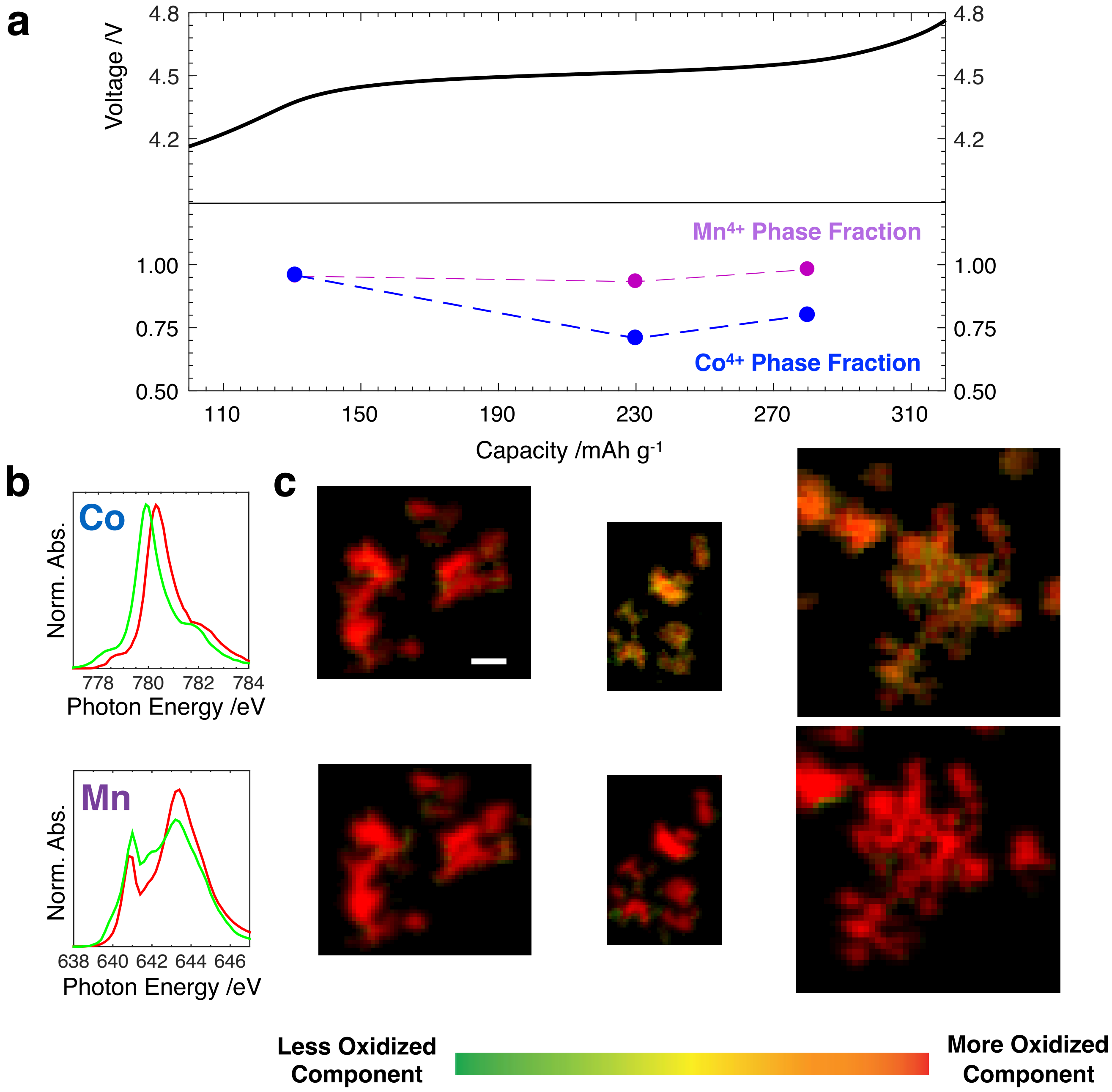

Supplementary Figure 4. Spatial Dependence of Mn and Co Spectroscopy during the Plateau. (a) The average Mn^{4+} and Co^{4+} phase fractions obtained by PCA for 3 *ex situ* STXM samples measured throughout the plateau, showing some reduction of the Co during the plateau. Since Co only makes up ~ 10% of the TM composition and is only reduced by ~ 0.25 e^- per atom, this change is relatively minor in comparison to the Ni and O changes during the plateau. (b) The principal components obtained from

PCA, which represent Co^{4+} (red), Co^{3+} (green), Mn^{4+} (red), and a mixture of Mn^{4+} and Mn^{3+} (green). (c) Spatial distribution of the components in (b), confirming no reduction of Mn during the plateau and that, as with the Ni, the reduction of Co is mostly confined to the particle surfaces. Scale bar is 500 nm.

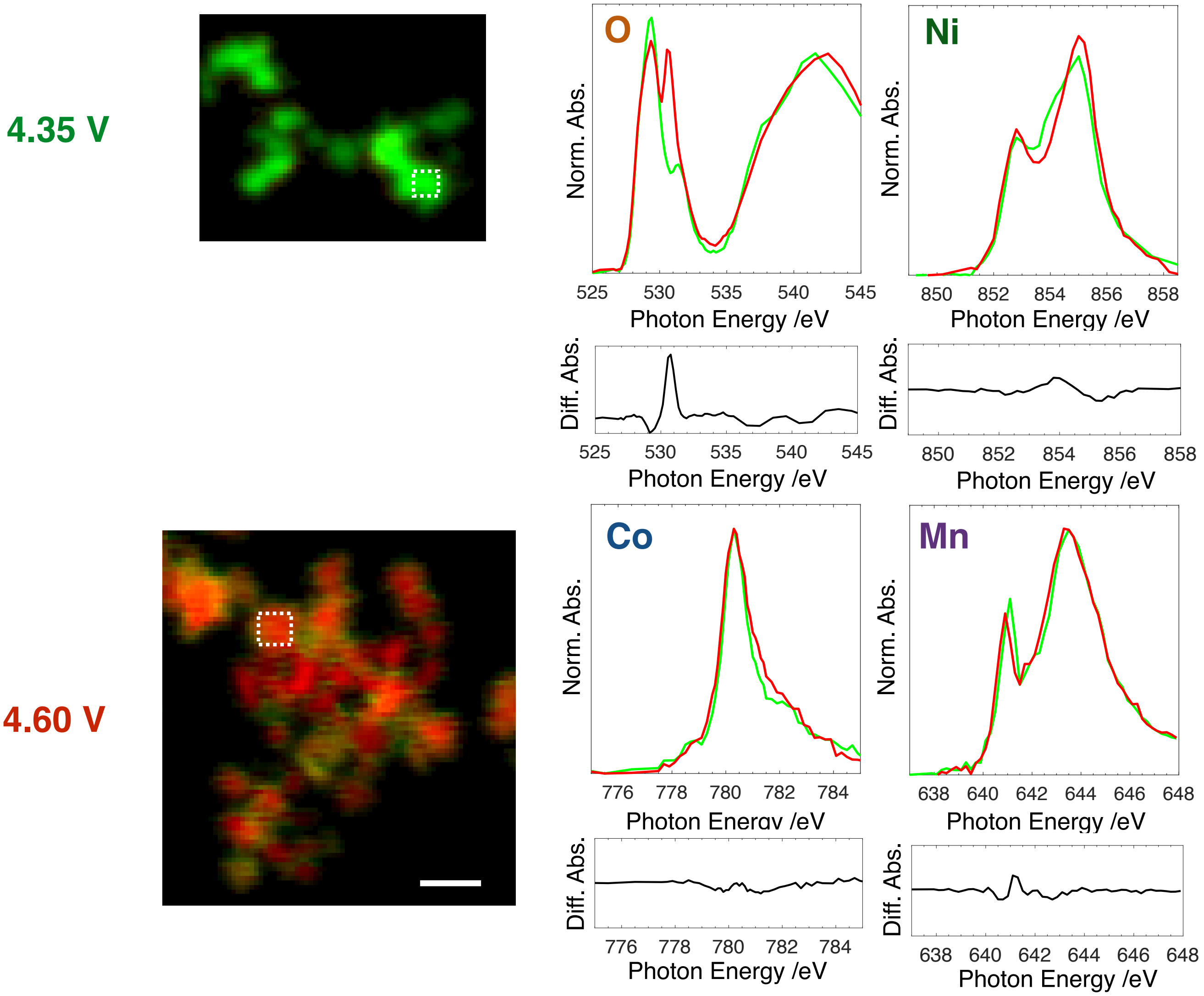

Supplementary Figure 5. Correlations in the Plateau Spectroscopy. Spectra obtained from the core of the two particles in the white boxes, one before the plateau at 4.35 V (green), and the other after the plateau at 4.60 V (red). Difference spectra are shown underneath in black. Color maps represent the distribution of the O spectra. There are some minor changes at the TM L_3 edges: the Ni edge changes shape slightly, the Co edge broadens, and the low energy peak at the Mn edge shifts by ~ 0.2 eV to lower energy. These may indicate some minor interactions between the TMs and the depopulated O_{2p} states;[8] however, these are subtle correlations and may be beyond the reproducibility limit of the STXM

measurement. Clearly the most significant change in the bulk is the growth of the new peak at 530.8 eV, providing strong evidence for O redox.

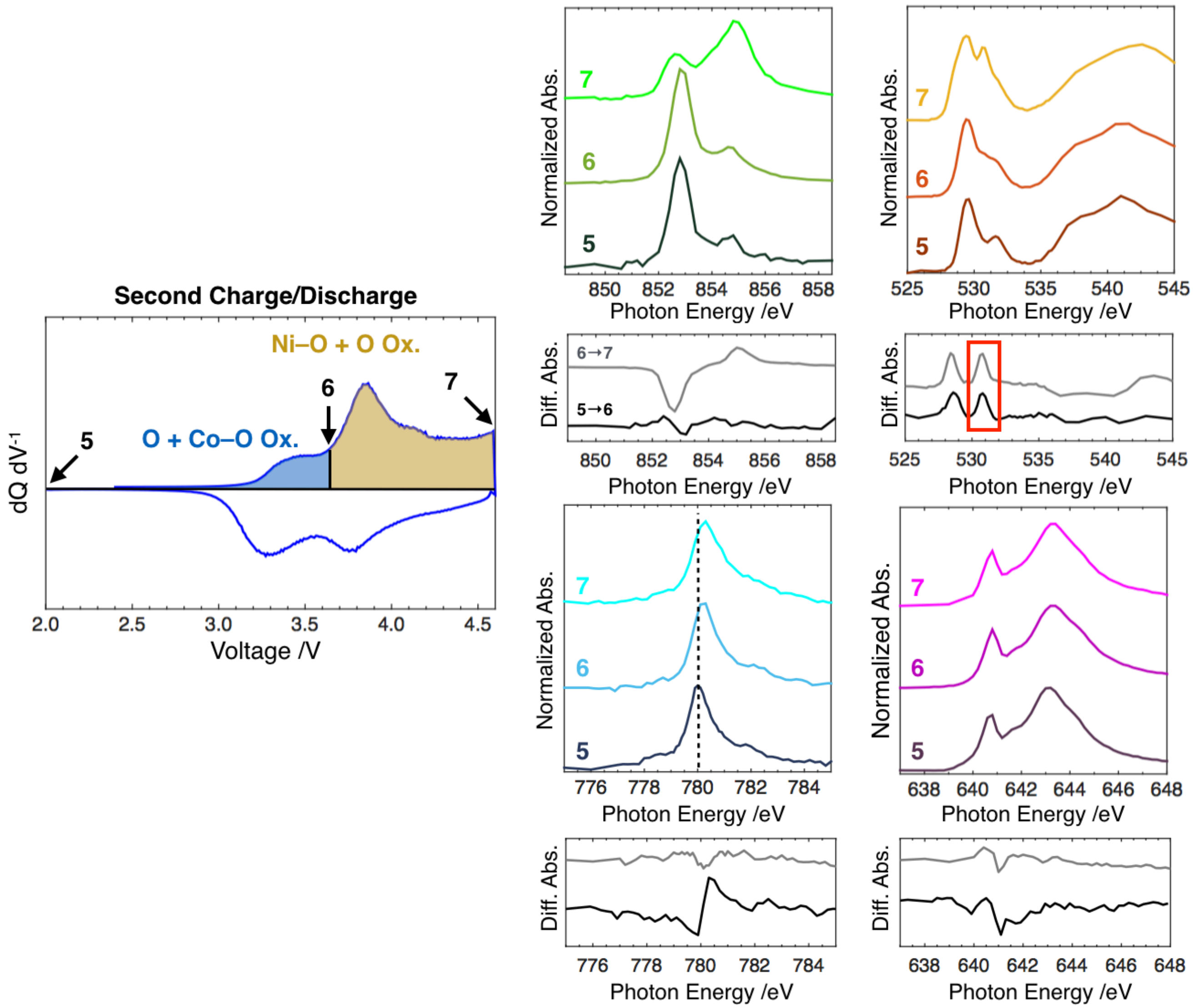

Supplementary Figure 6. Average Spectroscopic Behavior during the Second Charge. Average TM L_3 and O K edge XAS obtained from STXM during the second charge, showing that the charge capacity below 3.65 V is primarily O and Co–O oxidation, while the capacity above 3.65 V is both Ni–O and O oxidation. This further confirms that a significant fraction of oxygen redox remains shifted to below the TM–O redox after the first charge plateau.

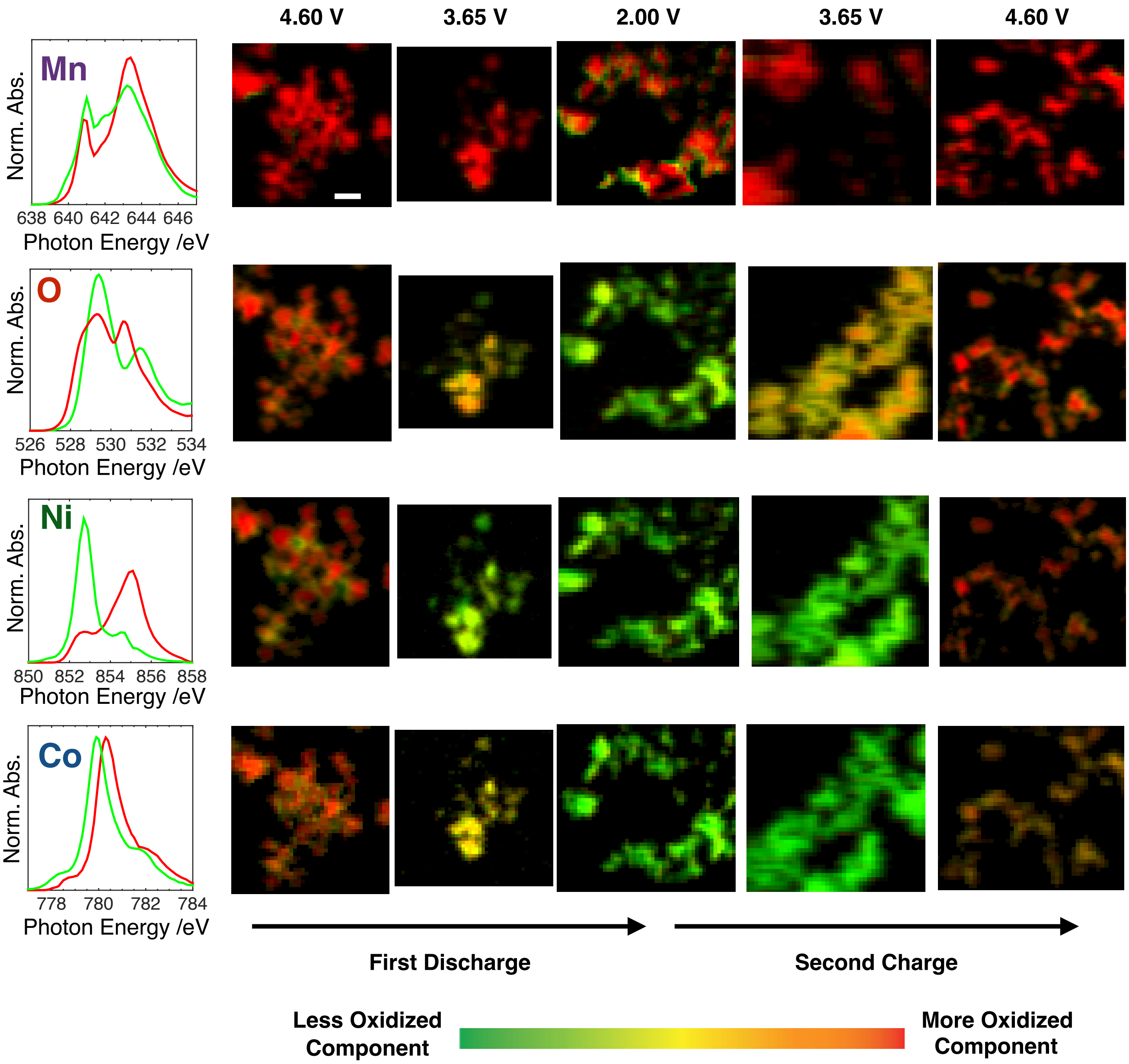

Supplementary Figure 7. Spatial Distribution of Redox Order Inversion after First Charge. Maps of the principal components obtained from PCA (left) performed on the first discharge and second charge at the TM L_3 and O K absorption edges. The Mn maps show the low voltage surface reduction during the first discharge, while the remaining edges show that the low voltage (< 3.65 V) capacity is primarily O redox. The Mn image at 3.65 V during the second charge was taken in a different region to the other edges.

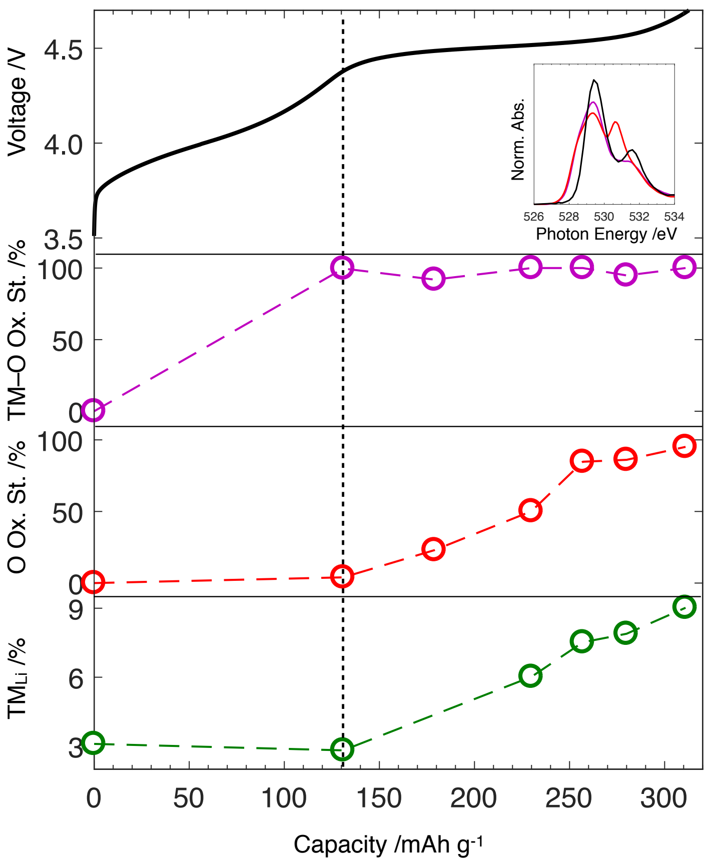

Supplementary Figure 8. Only O Redox is Linked to TM Migration. Plot of the TM–O (purple) and O (red) fractional oxidation states obtained from linear combination fitting of the end member spectra obtained from NMF (inset) to the average spectra versus the migrated TM fraction as a function of capacity. The TM–O oxidation state is calculated assuming that the red and purple end members have 100% TM–O oxidation, while the O oxidation state is calculated assuming only the red end member has 100% O oxidation.

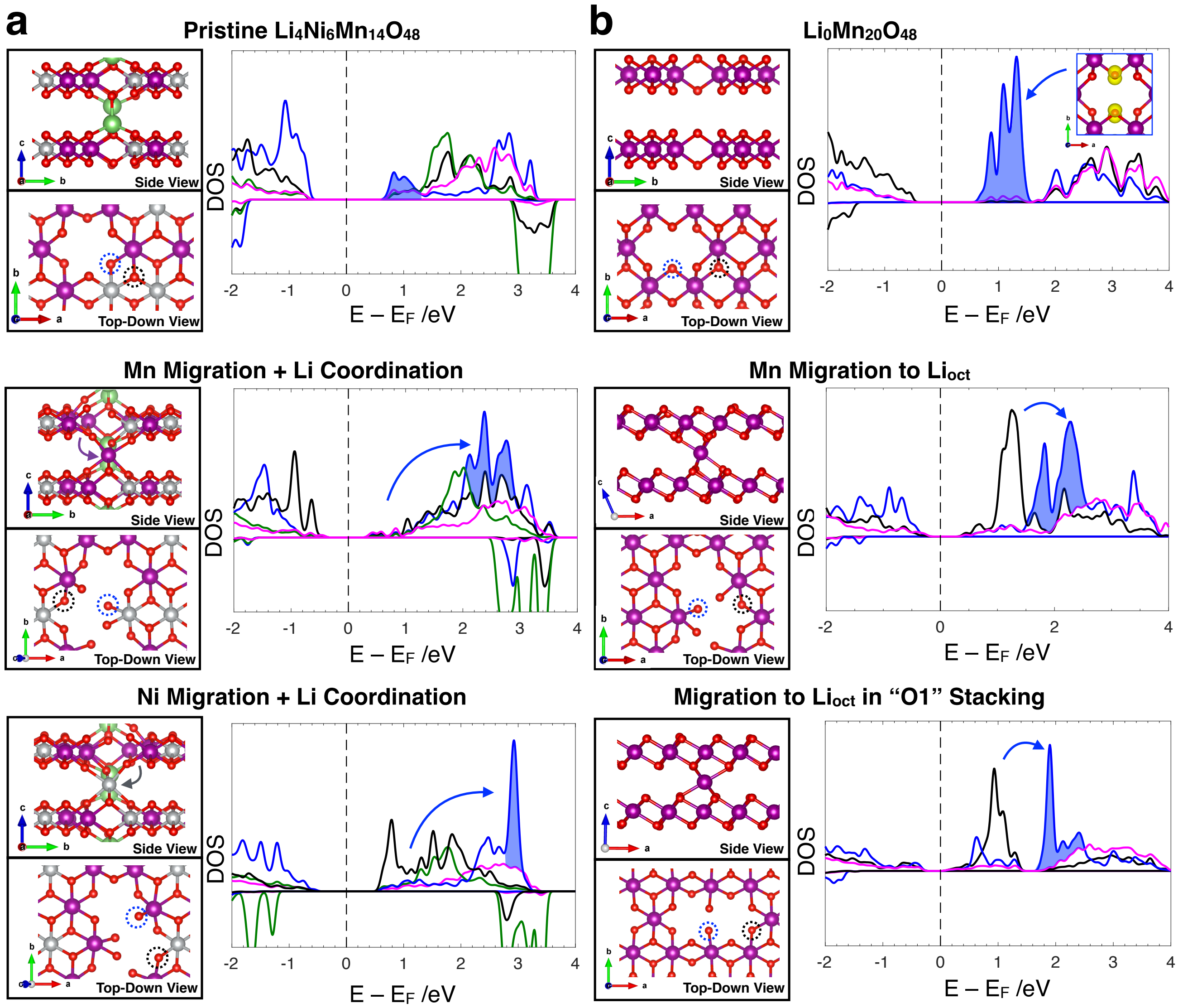

Supplementary Figure 9. DOS of other Migration Pathways. (a) DOS of a partially delithiated supercell before and after migration of Ni and Mn, with the decoordinated $O^{(1)}$ becoming additionally coordinated by a Li ion after TM migration. The results are largely similar to those of the fully delithiated supercell. (b) DOS of a fully delithiated Mn-only supercell before and after migration in O3 and O1 stacking configurations (see Methods). The shift of the $O^{(1)}$ states to higher energy is observed in both cases, though appears to be less than in the mixed Ni and Mn supercell.

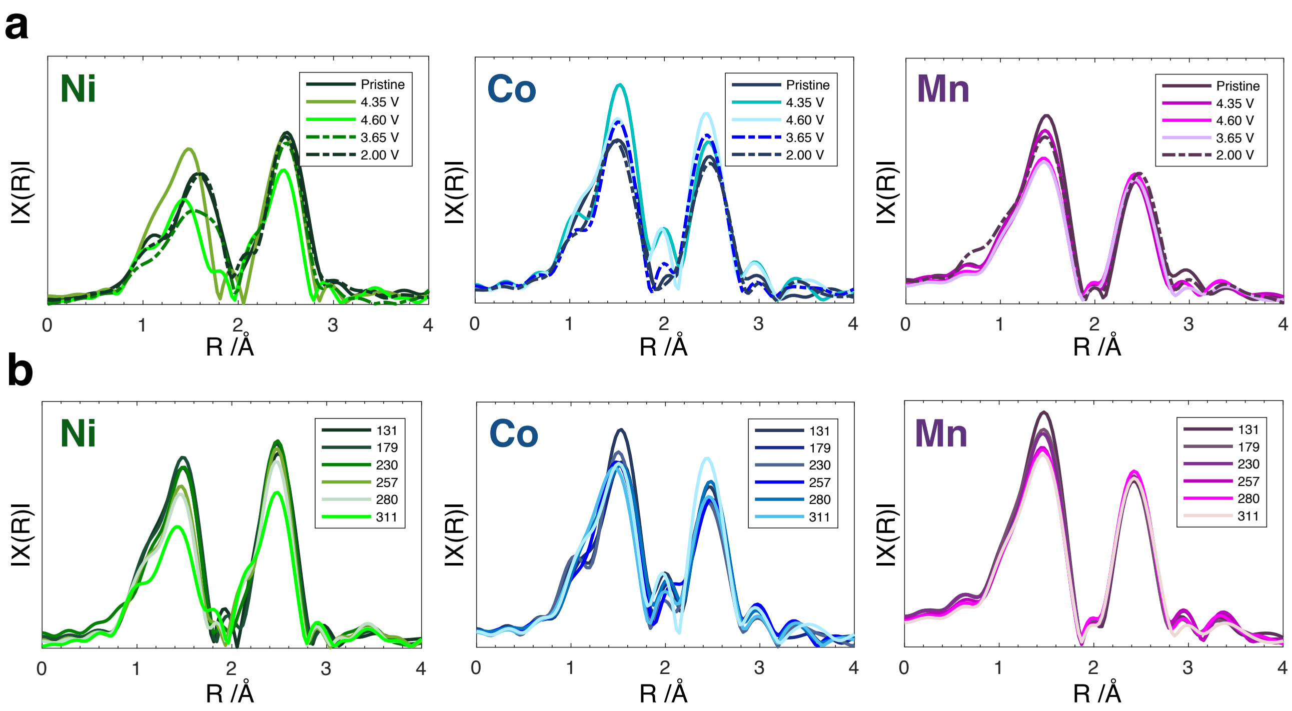

Supplementary Figure 10. First Cycle EXAFS. Radial distribution functions of the photoelectron scattering intensity obtained through EXAFS at the Ni, Co, and Mn K edges. (a) Data for the entire first cycle, and (b) Intermediate points throughout the plateau at the capacities specified in mAh g^{-1}. The large decrease in scattering intensity of both the first and second shell at the Ni K edge suggests that its local coordination environment changes substantially during the plateau, consistent with Ni being the primary migrating TM.

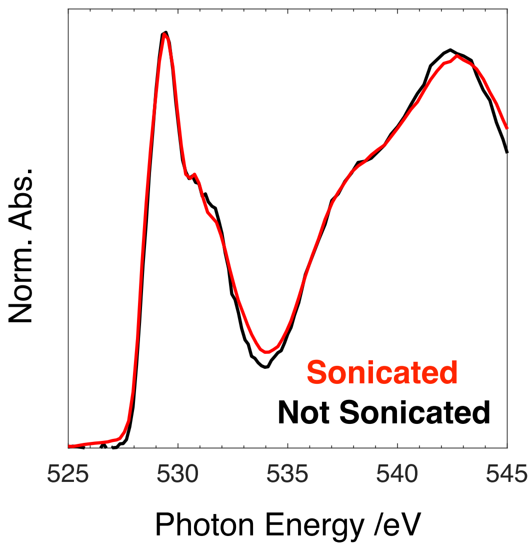

Supplementary Figure 11. Effect of Sonication on Surface Structure. TEY XAS acquired for the fully charged LMR-NMC electrode after sonication in DMC (i.e. mimicking the STXM sample preparation procedure) (red) and for the intact fully charged electrode (black).

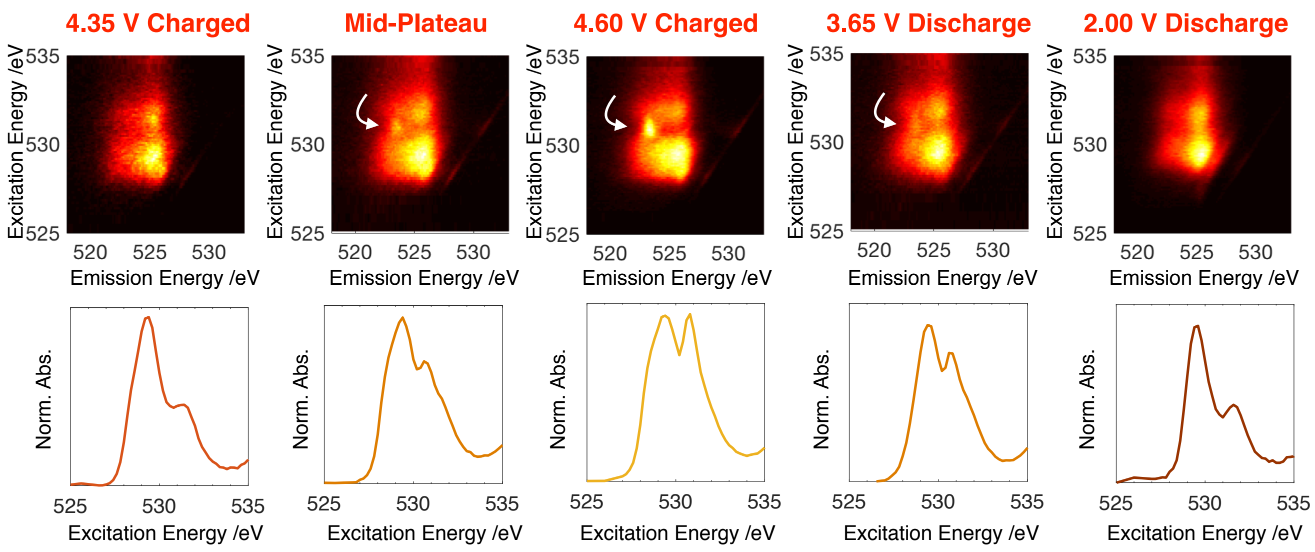

Supplementary Figure 12. Correlation between RIXS and STXM-XAS. Top: RIXS maps acquired throughout the first cycle, showing the gradual increase of the 523.75 eV emission feature during the voltage plateau and its persistence below 3.65 V on discharge. Bottom: STXM-XAS spectra at the same points showing the same trend for the 530.8 eV absorption feature. The "Mid-Plateau" sample was harvested at a first-charge capacity of 230 mAh g^{-1}.

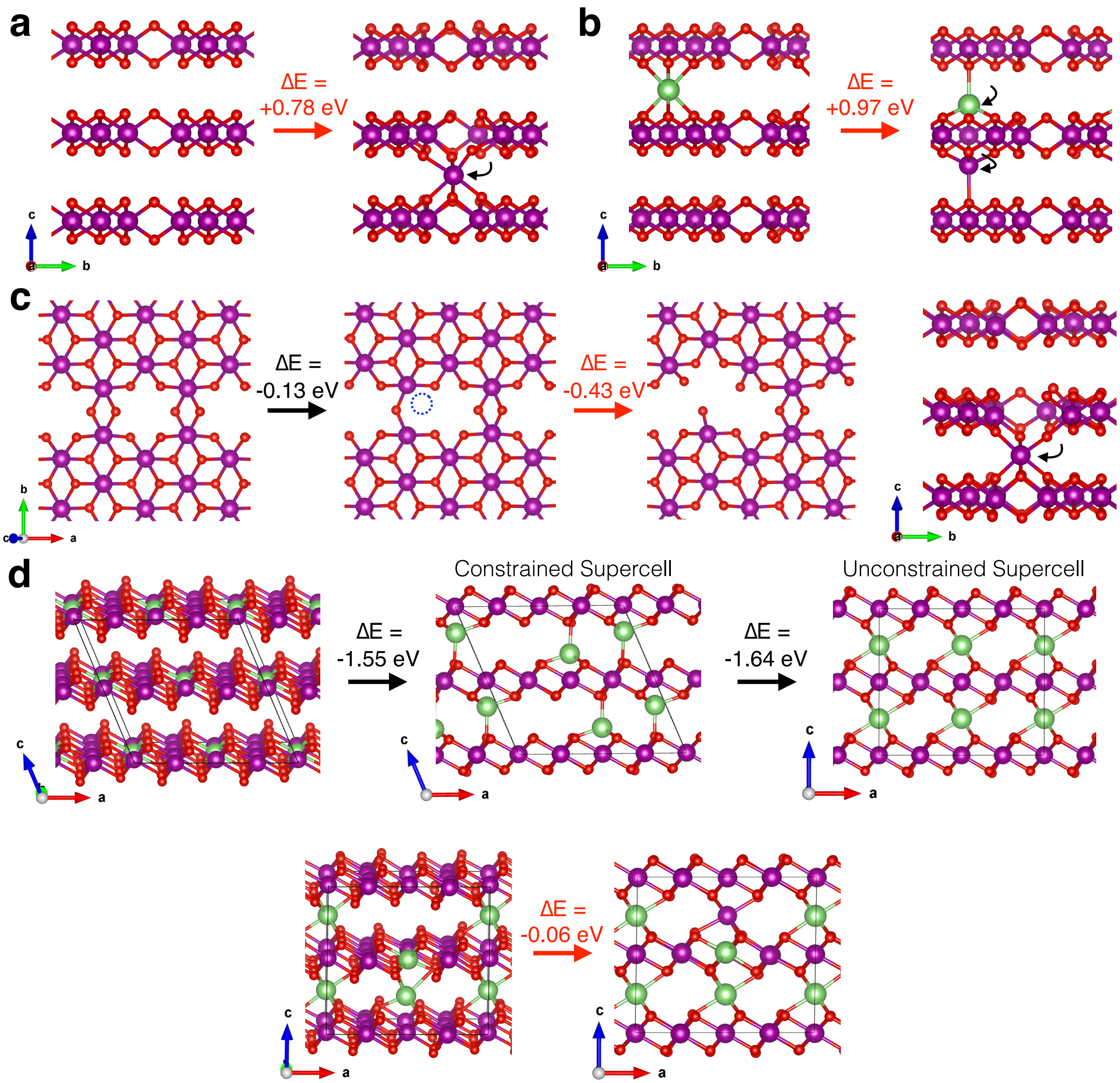

Supplementary Figure 13. Schematics of the Migration Pathways Investigated in this Study. (a) TM migration only, (b) TM migration coupled with Li migration into the newly stable tetrahedral site above the TM vacancy, (c) TM migration in the presence of an oxygen vacancy, (d) TM migration coupled with a change in stacking order. The stacking order change occurs with no energetic barrier when the lattice parameters are unconstrained and allowed to relax. The red arrows indicate the TM migration

steps. Other steps are prerequisites to a particular migration pathway. All steps have their associated enthalpies indicated as ΔE.

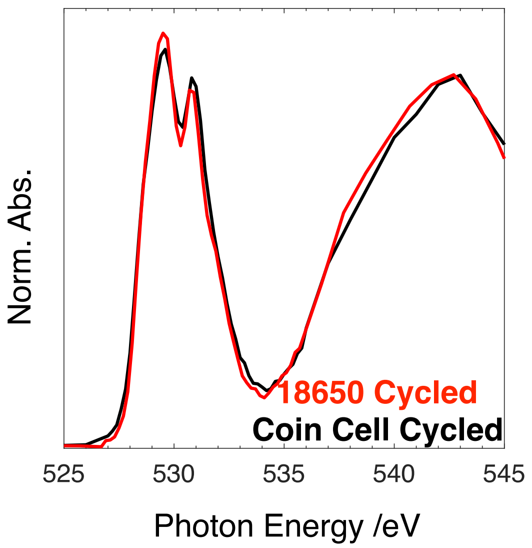

Supplementary Figure 14. Comparison of Electrodes Prepared in 18650 Cells and Coin Cells. STXM-XAS of electrodes at the end of the second charge having undergone the first full cycle in an 18650 cell (red) and a coin cell (black). The 18650 electrode was dismantled in a dry room after the first cycle and then shipped to Stanford before undergoing the second cycle, replicating the procedure for the 501st cycle sample shown in Figure 5 in the main text. The coin cell sample was dismantled in an argon filled glovebox only.

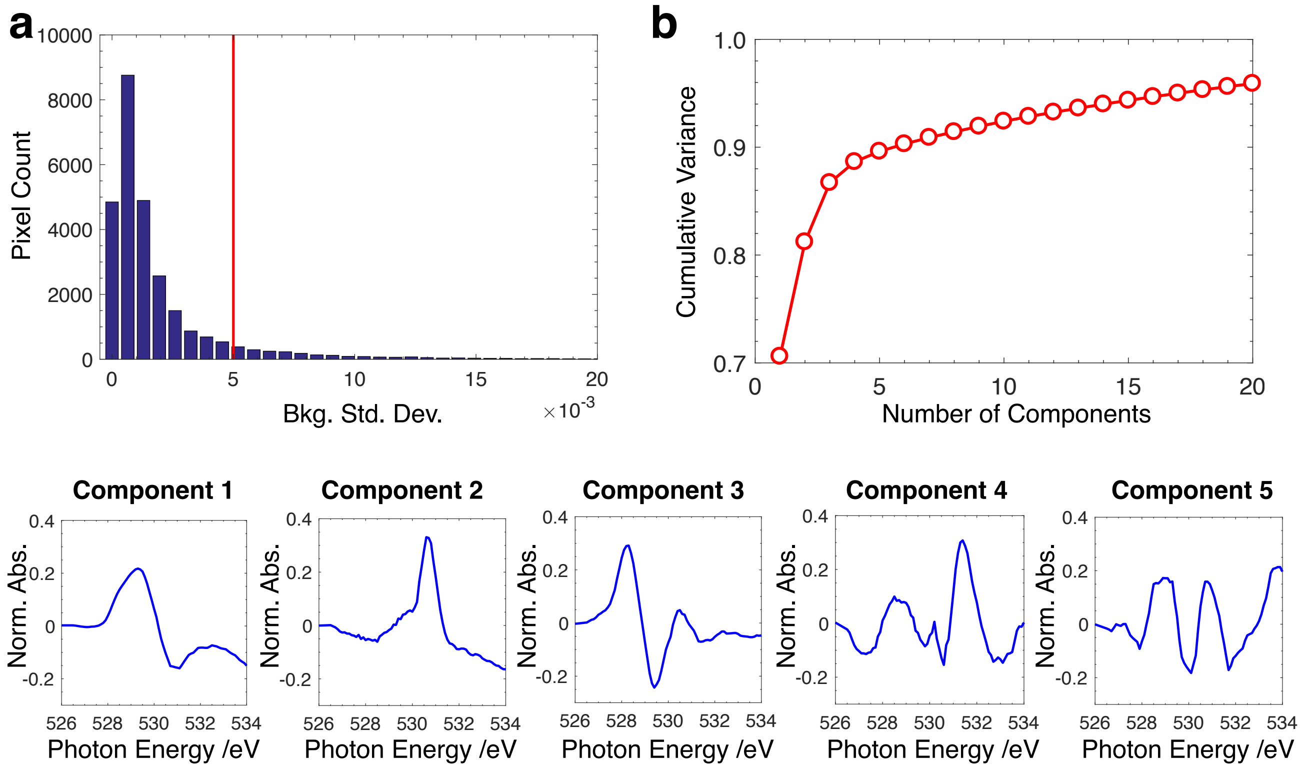

Supplementary Figure 15. PCA Summary. (a) Histogram of the standard deviation in the background region for the oxygen spectro-images taken during the plateau. (b) Cumulative variance of the first 20 principal components obtained by PCA. (c) The first five principal components.

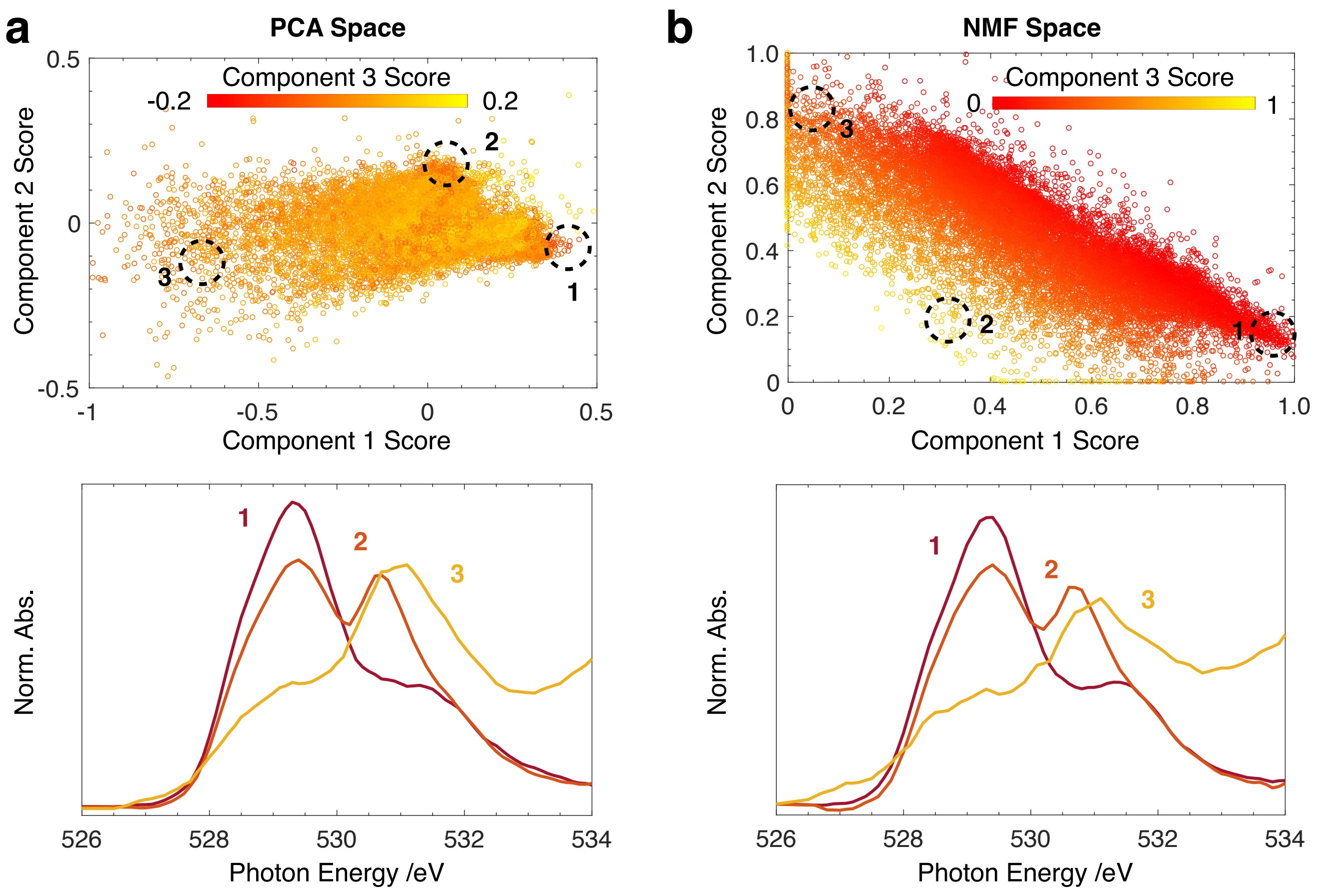

Supplementary Figure 16. Comparison of PCA and NMF Methods. (a) Top: plot of the plateau STXM data in PCA space using the first three principal components; Bottom: the end-member spectra corresponding to the select regions shown. (b) The corresponding plots in NMF space.

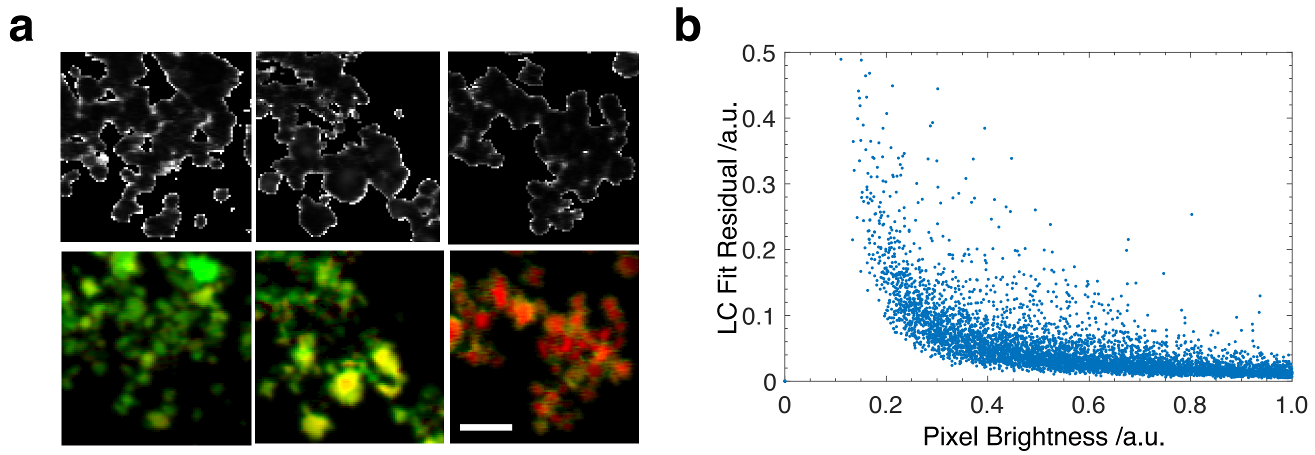

Supplementary Figure 17. Evaluation of End-Members Identified by NMF. (a) Residual maps for the three O spectral maps shown in main text Figure 4c. In the greyscale residual maps, white corresponds to a residual of 0.25 or higher and black corresponds to 0. (b) Plot of the single pixel residual vs pixel brightness for the entire plateau dataset. The brightness is linearly proportional to the integrated post-edge area of the single pixel spectrum.

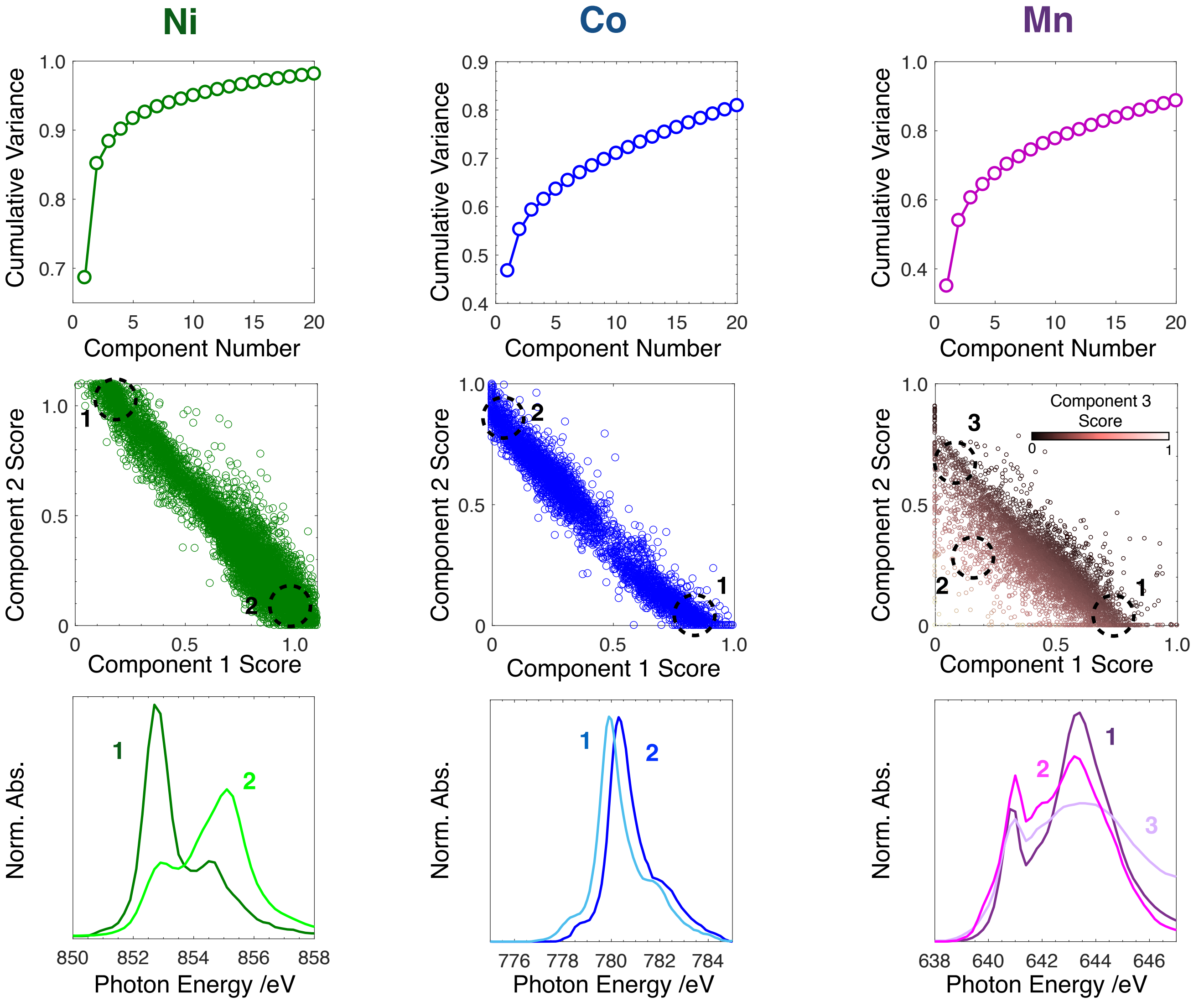

Supplementary Figure 18. PCA/NMF Results at the TM L_3 Edges. Top: PCA cumulative variance as a function of number of components. Middle: Plots of the data in NMF space with the indicated number of components (2, 2, and 3, for Ni, Co, and Mn, respectively). Bottom: the end-member spectra corresponding to the extreme points selected in the NMF-space plots.

Supplementary Tables

Supplementary Table 1. *C2/m* Crystallographic Structure File Used for Rietveld Refinement of Pristine Material Pellet. "TM" refers to a combination of Ni, Co, and Mn approximately equal to the bulk composition of 0.21 : 0.08 : 0.54, respectively.

a = 4.9558(1) Å b = 8.5745(13) Å c = 5.0316(1) Å ß = 109.267(1)˚

**Site**	**Occupancy**	**x**	**y**	**z**
2*c*	98.2 % Li, 1.8 % Ni	0	0	0.5
4*h*	98.2 % Li, 1.8 % Ni	0	0.6667	0.5
2*b*	18.8 % Li, 81.2 % TM	0	0.5	0
4*g*	18.8 % Li, 81.2 % TM	0	0.1600	0
4*i*	100 % O	0.2394	0	0.2242
8*j*	100 % O	0.2394	0.3333	0.2242

Supplementary Table 2. Rietveld Refinement Results. Rietveld refinement results in the *C2/m* space group for the first cycle samples used in this study. "c1" and "d1" indicate samples harvested during the first charge and discharge, respectively. The three samples defined by a capacity cut-off are the intermediate plateau samples. The values in parentheses under each sample represent the assumed amount of Li in the Li and TM layers, respectively, based on an earlier neutron diffraction study.[9] The total TM stoichiometry is 0.83; thus the percentage of TM in the Li layer is the TM_{Li} occupancy divided by 0.83.

Sample	a /Å	b /Å	c /Å	ß /°	Oxygen *z* Position	TM_{Li} Occupancy	R_{wp}
Pristine (Li 1+0.17)	4.9554(1)	8.5750(2)	5.0310(1)	109.267(1)	0.2329	0.0257(8)	1.92
c1_4.35V (Li 0.575+0.17)	4.9222(2)	8.5249(3)	5.0616(2)	109.103(2)	0.2329	0.0234(14)	7.71
c1_230mAh/g (Li 0.287+0.14)	4.9219(2)	8.5262(3)	5.0884(2)	109.055(2)	0.2329	0.0500(16)	7.28
c1_257mAh/g (Li 0.217+0.12)	4.9240(2)	8.5300(3)	5.0884(2)	109.079(3)	0.2328	0.0624(18)	6.72
c1_280mAh/g (Li 0.163+0.10)	4.9249(2)	8.5323(3)	5.0871(2)	109.096(2)	0.2283	0.0655(15)	7.06
c1_4.60V (Li 0.085+0.085)	4.9222(3)	8.5308(4)	5.0649(1)	109.177(3)	0.2308	0.0749(15)	7.69
d1_3.65V (Li 0.575+0.085)	4.9499(1)	8.5671(2)	5.0902(2)	109.094(2)	0.2323	0.0583(11)	4.43
d1_2.00V (Li 0.985+0.085)	4.9698(1)	8.6035(2)	5.0573(1)	109.211(1)	0.2330	0.0391(5)	4.00

Supplementary Table 3. Rietveld Refinement Results with Variable Oxygen Content. Refinement as in Supplementary Table 2, but with the oxygen site occupancy included as a variable. The refined oxygen content varies significantly and unrealistically due to the insensitivity of X-rays to light elements such as oxygen. Nonetheless, the refined TM_{Li} values are very similar to those obtained when the oxygen content is fixed at 1, and the overall trend is conserved.

**Sample**	**a /Å**	**b /Å**	**c /Å**	**ß /°**	**Oxygen *z* Position**	**Oxygen Site Occ.**	**TM_{Li} Occ.**	**R_{wp}**
Pristine (Li 1+0.17)	4.9552(1)	8.5751(2)	5.0309(0)	109.266(1)	0.2330(3)	**0.9600(4)**	**0.0306(30)**	2.42
c1_4.35V (Li 0.575+0.17)	4.9216(2)	8.5250(3)	5.0621(2)	109.113(2)	0.2342(3)	**0.9645(3)**	**0.0308(14)**	7.72
c1_230mAh/g (Li 0.287+0.14)	4.9209(2)	8.5263(3)	5.0881(2)	109.059(2)	0.2337(3)	**0.9116(4)**	**0.0477(9)**	7.12
c1_257mAh/g (Li 0.217+0.12)	4.9232(2)	8.5311(3)	5.0895(2)	109.082(3)	0.2324(4)	**0.8727(4)**	**0.0677(10)**	6.47
c1_280mAh/g (Li 0.163+0.10)	4.9242(2)	8.5332(3)	5.0881(2)	109.108(3)	0.2288(3)	**0.8581(4)**	**0.0665(9)**	6.65
c1_4.60V (Li 0.085+0.085)	4.9206(2)	8.5321(4)	5.0647(1)	109.183(3)	0.2326(4)	**0.8434(4)**	**0.0747(11)**	7.69
d1_3.65V (Li 0.575+0.085)	4.9469(1)	8.5716(2)	5.0930(1)	109.115(1)	0.2359(2)	**0.8753(3)**	**0.0458(6)**	4.02
d1_2.00V (Li 0.985+0.085)	4.9697(1)	8.6038(2)	5.0573(1)	109.217(1)	0.2337(2)	**0.945(2)**	**0.0393(4)**	3.82

Supplementary Notes

Supplementary Note 1. Discussion of RIXS Features

The features revealed in our RIXS maps can be understood in three parts. First, the broad vertical feature with excitation energy (vertical axis) above 535 eV corresponds to excitation to the itinerant (broad) hybridized TM_{4sp}–O_{2p} band. The emission feature is mostly from a direct decay process of oxygen valence band electrons to the O-*1s* core holes. Second, the two packets of intensity at the same emission energy (horizontal axis) of about 522-526 eV with excitation energy below 535 eV is due to the same decay process but after excitation into the TM-O hybridized states. This can be clearly seen in the alignment of the excitation energies of these two features align with the two hybridized TM–O antibonding features in the pre-edge of the XAS. The 522-526 eV emission features have been studied extensively and are ubiquitous in transition-metal oxides.[10,11] Third, a striking feature at 530.8 eV excitation energy and 523.75 eV emission energy emerges between 4.35 – 4.60 V and disappears upon discharge. RIXS maps collected at intermediate points throughout the plateau and discharge (Supplementary Fig. 12) show that this feature behaves exactly the same as the oxygen redox feature observed in transmission XAS, emerging gradually during the charge plateau and persisting below 3.65 V during discharge. Therefore, although RIXS has a probing depth similar to FY-XAS (~100 nm at the O K edge), the resolved emission energy provides enhanced sensitivity to specific bulk states. By further correlating with STXM-XAS, we confirm that the sharp feature at 523.75 eV emission energy has exactly the same excitation energy of the bulk oxygen redox feature at 530.8 eV.

The narrowness of the 523.75 eV emission feature is also notable, and may indicate a certain degree of localization of the depopulated O_{2p} states. However, understanding whether this necessarily

means that they are localized in space or just form a narrow band requires a thorough computational study that is beyond the scope of this work.

Supplementary Note 2. First-Principles Methodology

We employ a supercell of $Li_{28}Mn_{14}Ni_6O_{48}$ (or equivalently $Li[Li_{0.17}Mn_{0.58}Ni_{0.25}]O_2$) which contains a similar amount of Li in the TM layer as our experimental material ($Li[Li_{0.17}Ni_{0.21}Co_{0.08}Mn_{0.54}]O_2$). The supercell consists of two TM layers and two Li layers, and ensures that the migrated TM defects are approximately 1 nm apart, limiting defect-defect interactions. Both of these structures contain two main types of oxygen environment: two-coordinate and three-coordinate ($O^{(1)}$ and $O^{(2)}$ in Figure 6b, respectively). While there is a debate as to the intermediate range clustering of the TMs in LMR-NMC (i.e. whether it is a nano-composite of monoclinic Li_2MnO_3 and rhombohedral NMC domains,[12] or a uniform solid solution monoclinic structure[13]), we note that in both cases there are only the two types of oxygen environment mentioned, and thus the effect of TM migration on anion redox in either case can be understood by observing the effect on $O^{(1)}$ and $O^{(2)}$.

We first investigate the effect of migration of a Ni or Mn ion, as these appear to migrate the most based on the significant changes to their EXAFS radial distribution functions during the 4.5 V plateau in Supplementary Fig. 10. Computational studies have shown similar migration pathways to be feasible in Li_2MnO_3.[14,15] As shown in Figure 6b and discussed in the main text, in the fully delithiated state this causes a substantial shift of the depopulated $O^{(1)}$ states to higher energy, rationalizing the various experimental observations presented in this work. The shift due to Ni migration is larger, and we find this is coupled with a more substantial distortion in the anion sublattice and the resulting Mn–O distances.

Of course, in reality the material is not fully delithiated and Li ions could form additional interactions with the de-coordinated oxygen ions. To investigate this effect, we show in Supplementary Fig. 9a the DOS of the same migrated structures but with some finite Li content, and with a concerted migration of the Li to re-coordinate the single-coordinate $O^{(1)}$ ion. While the overall shift of the

depopulated O_{2p} states is slightly lower, clearly the effect of the lost TM coordination partner is much greater, and the O_{2p} states still invert order with the Ni_{3d} redox states.

While we observe the TMs to migrate experimentally, identifying the most prevalent migration pathways that occur in practice requires exploring a vast configurational space, considering in-plane and stacking order, local composition, TM oxidation state, Li content, and anion and cation vacancies.[14,16-18] We intend to show here only the inevitable consequence of TM migration, which we know to occur experimentally, on O redox activity. Thus, rather than explore this large space, we use a simplified structure containing only Mn ($Li_{28}Mn_{20}O_{48}$ or $Li[Li_{0.17}Mn_{0.83}]O_2$) to explore four different TM migration mechanisms of varying favorability and establish how general our observations are. The four migration mechanisms are: (a) Mn migration only; (b) Mn migration coupled with Li migration into the newly stable tetrahedral site above the Mn vacancy; (c) Mn migration in the presence of an oxygen vacancy; (d) Mn migration coupled with a change in stacking order. Supplementary Fig. 13 shows the migration scheme and favorability for each mechanism. We explored migration of Mn from two different sites in the TM layer (neighboring two LiO_6 and four MnO_6 octahedra or surrounded by six MnO_6 octahedra) into adjacent octahedral or tetrahedral sites in the Li layer. Out of the four migration classes, only (c) and (d) exhibited energetic favorability for the pathways investigated. However, in all cases we observed the same shifting to higher energy of the de-coordinated O_{2p} states, confirming that the change in local coordination is the dominant factor affecting the O redox chemistry. The DOS of two representative migration pathways are shown in Supplementary Fig. 9b. We discuss the likelihood of the two favourable migration classes below.

(c) While we observe that oxygen evolution is most prevalent at the primary particle surfaces, it is possible that a low concentration of oxygen vacancies could exist in the bulk and be below the detection limit of XAS and XRD. Indeed, only ~ 2.5 % oxygen vacancies could account for one vacancy per TM

migration event during the plateau, and would result in a nominal reduction of the Mn oxidation state on discharge by only ~ 0.2 *e*. Supplementary Fig. 13 shows that oxygen vacancy formation at the oxygen sites coordinated to two Mn ions is enthalpically favored by 0.13 eV, consistent with previous reports.[19] As discussed in the main text, this is only one of a variety of possible redox active oxygen sites and does not therefore imply that all oxygen redox in LMR-NMC is intrinsically unstable. Supplementary Fig. 13 shows that Mn ions neighboring the vacancy favorably migrate.

(d) Under certain conditions, we found a change in stacking order of the oxygen sub-lattice to occur with no energetic barrier. Supplementary Fig. 13 shows that when the Li sites in the TM layer line up in adjacent layers, an "O1" oxygen stacking order (i.e. ABAB rather than the ABCABC or "O3" order of the pristine material) is spontaneously adopted with significant stabilization. Stacking order changes have been observed experimentally in other classes of well-ordered Li-rich materials.[20] However, the structural prerequisite is likely to only occur in nano-scale domains within a single LMR-NMC crystal given the substantial degree of stacking faults and in-plane disorder they exhibit.[13] Nonetheless, TEM studies of the fully charged material have shown a patchwork structural evolution,[21] which could reflect local stacking order changes. Supplementary Fig. 13 shows migration in the O1 stacking regime to be favorable when a Li ion occupies the octahedral site directly below the migrating TM.

Future work could explore the effect of Li content, TM composition, and TM configuration on the overall favorability of the various pathways. The entropic contribution to the migration free energy would also be an interesting avenue of further study, as we expect the increased structural disorder brought about by TM migration should further favor it.

Supplementary Methods

Figure 5b Electrochemical Measurements and Electrode Harvesting

The voltage curves in Figure 5b were measured for commercially prepared 18650 electrodes (Samsung) containing 92% LMR-NMC and 4% carbon and 4% binder composite by mass. These electrodes were assembled into 18650 cells with graphite negative electrodes and a 1.3 M $LiPF_6$ in 3:8:9 (vol/vol/vol) fluoroethylene carbonate (FEC)/hydrofluoro ether (HFE)/DMC electrolyte with some proprietary additives, and underwent the first formation cycle at 45 ˚C at C/5. The "2nd cycle" sample in Figure 5b was dismantled after the formation cycle, while the "501st cycle" sample was dismantled after 500 cycles at a 1C/2C charge/discharge rate between 2.5 and 4.55 V, with a constant voltage hold at 4.55 V until C/20 current was reached. The cells were dismantled in the discharged state in a dry room and the harvested electrode sheets were packaged under dry air and shipped to Stanford, where they were stored in an argon filled glove box. Supplementary Fig. 14 shows that this has no effect on the O K edge spectrum as compared to the normal coin cell dismantling procedure, indicating that dismantling in a dry room in the discharged state does not affect the material structure. 9mm diameter discs were punched from these sheets and assembled into coin cells as described above, with the exception of the electrolyte being the proprietary electrolyte they were initially cycled in. The cells were first discharged to 2.5 V with a constant voltage hold until 2.4 mA g^{-1} was reached. The voltage curves at 4 mA g^{-1} were then measured.

For *ex situ* sample preparation, cells were charged at 24 mA g^{-1} (~ C/11) to the indicated voltage and held until a current density of 2.4 mA g^{-1} (~C/110) was achieved. Samples that are defined by a capacity cut-off (e.g. at intermediate points during the plateau) were not held at a voltage but were rested (open circuit) for 30–120 minutes and after reaching the desired capacity at 24 mA g^{-1}. Cells were dismantled using a coin cell decrimper (MTI Corporation). The electrodes were then washed with excess

DMC and dried under vacuum. Samples were transferred to the desired instrument in a double-sealed aluminium pouch to prevent air exposure.

Rietveld Refinement

We used the TOPAS software package (Academic v6) for Rietveld refinement. In the first stage, Pawley fitting was employed to find the best space group for refinement. We found the C2/m space group to give the best results, consistent with previous reports on similar Li-rich layered materials.[13,22,23] We assumed that Ni, Co, Mn, and Li atoms were evenly distributed through the 4g and 2b sites in the TM layer, and migrated transition metal from 4*g* and 2*b* sites was assumed to redistribute equally to the 2c and 4h sites in the Li layer. In all refinement, all lattice parameters were allowed to vary in addition to the oxygen *x* and *z* positions and the *y* positions of the 4*g* and 4*h* sites. Supplementary Table 1 shows the crystallographic structure file used for Rietveld refinement of the pristine XRD pattern. For intermediate states of charge, we assumed the relative ratio of Li metal in the TM layer and Li layer according to a previous report, which refined Li occupancies using neutron diffraction.[9] Supplementary Table 2 shows the Rietveld refinement results for all samples used in this study.

To confirm the robustness of the observed TM migration trend, we also performed a parallel refinement with variable oxygen content. Although we only see evidence through STXM of oxygen loss and reconstruction in small near-surface domains, including this additional variable reveals whether it may be correlated with the refined TM_{Li} fraction and therefore influencing the observed trend. Supplementary Table 3 shows the full refinement results when oxygen vacancies are included as a variable. The trend in the refined TM_{Li} fraction is almost identical to Supplementary Table 2, where the oxygen site occupancy was fixed at 1. When the oxygen content is allowed to vary, it does so significantly, falling by over 15% during the plateau before returning to close to 1 at the end of discharge.

These large swings are unphysical – requiring reversible oxygen exchange with the electrolyte – and are likely due to the insensitivity of X-rays to light elements such as oxygen, which makes probing oxygen content through XRD extremely unreliable. However, the analysis confirms that the observed TM migration trend is largely unaffected when oxygen vacancies are included in the refinement, confirming that it is a reliable and robust observation. While the inclusion of oxygen vacancies slightly improves the quality of the fit defined by R_{wp}, this is likely just a consequence of including more fitting variables. Since this model does not give meaningful results, we use the model that assumes full oxygen site occupancy, as the reduced number of variables improves the robustness and stability of the fit.

STXM Data Processing and Principle Component Analysis

To obtain absorbance data from the raw transmitted spectra, a cluster of single pixel spectra from a region in each STXM image containing no sample were averaged to obtain the background I_0 spectrum. This was then applied to every pixel in the rest of the STXM spectro-image through $-\ln(I/I_0)$, where I is the single pixel spectrum, to obtain absorbance spectro-images.

To obtain average absorption spectra, the aligned absorbance images were filtered at an energy of non-zero intensity for the LMR-NMC (typically 529.5 eV, 644 eV, 780 eV, and 852 eV for the O K, Mn L, Co L, and Ni L edges, respectively). A threshold was set for the intensity of this energy relative to the background intensity, and the pixels below this threshold were set to zero. The remaining un-normalized pixels were then summed to yield the average spectrum. For presentation, normalization of the average spectra was done by subtracting the background intensity and then dividing by the integrated area of either the full L_3 edge or, in the case of oxygen, the post edge area.

In order to systematically detect and map the key underlying spectral changes in the STXM data, we perform principal component analysis (PCA). We use the oxygen spectro-images collected *ex situ*

throughout the plateau (Figure 4 in the main text) as a representative example to explain our methodology. The data is preprocessed by compiling every pixel of the spectro-images taken at each condition along the plateau (i.e. charged to 131, 179, 230, 257, 280, and 311 mAh g^{-1}) into one large matrix of ~ 74,700 pixels x 50 energy points (spanning the 520 – 535 eV pre-edge range). Offsets due to variations in the X-ray energy calibration between experiments were accounted for using a reference sample, usually the pristine LMR-NMC powder. Each pixel was normalized by subtracting the mean background (520 – 526 eV) intensity and then dividing by the integrated pre-edge (525 – 535 eV) area. While this is technically not a true normalization, it functions to normalize pixels containing the same underlying chemical states but with different overall intensities (due to thickness variations), which is sufficient for any linear trend to be decomposed through PCA. Thus, this served as a good approximate normalization range without requiring the measurement of substantially more data beyond the pre-edge, which is experimentally time-consuming in STXM. In any case, we found PCA and NMF to be relatively insensitive to the normalization procedure, though it sometimes facilitated identifying end-members.

After normalization, the data was filtered by removing points with relatively large amounts of noise (as measured by standard deviation) in the background region (520 – 526 eV). This serves to remove pixels that originally had very low signal but were amplified by the normalization. The distribution of noise in the pre-edge is shown in the histogram in Supplementary Fig. 15a. The red line indicates the noise cutoff beyond which we no longer include the points in our analysis; this process removed approximately 10% of the data. The data was then mean-centered at each energy.

We then performed PCA on the data using the singular value decomposition (SVD) method.[24,25] Supplementary Fig. 15b shows the cumulative variance explained by the first 20 principal components identified by PCA. The most obvious gains in cumulative variance are seen for the first three components, which explain 87% of the total variance in the data. Furthermore, Supplementary Fig. 15c

shows that the 4th and 5th components exhibit a high degree of redundancy with the first three but appears to contain more information than just noise. This could be due to experimental limitations such as small energy shifts between the samples used that aren't adequately accounted for by the reference correction (e.g. due to intra-experiment drift in energy calibration). As described later, including more than 3 components did not yield meaningful improvement in the analysis. We therefore conclude that the remaining variance beyond 87% is mostly noise and that the data can be adequately described by the first three principal components. This serves to reduce the dimensionality of the data from [x, y, 50] to [x, y, 3].

Once we have identified the principal components, it is typical to project the data into the lower-dimensional PCA space to identify the extreme points (i.e. the end member spectra), as shown in Supplementary Fig. 16a. We average a small cluster of the observed single pixel spectra at the extremes that bound the data in PCA space to find the true end member spectra. Often non-negative matrix factorization (NMF) facilitates this analysis. Since NMF is constrained to non-negative combinations of non-negative components, in applications to spectroscopy this can generate components that more closely resemble the real varying components of the system and thereby make identifying extreme points easier, as shown in Supplementary Fig. 16b. In this case, while acceptable end-members can be identified in PCA space, they are more clearly visible in NMF space. These end members represent the reduced LMR-NMC spectrum (1), the oxidized LMR-NMC spectrum (2), and the carbon/binder matrix (3). We note that these end members are not necessarily pure spectral components, and may be a combination of more than one pure spectral component that happen to vary together. However, being end-member spectra by construction, they adequately represent the spectral variation in the data and are therefore useful in mapping the spatial distribution of chemistry in LMR-NMC.

We then perform a non-negative linear least squares regression on every normalized pixel to calculate the fraction of each end member present. Total end member fractions for a given sample are determined by a linear combination fit of the end members to the average spectrum obtained by summing all the pixels as for Figure 2b-e in the main text. A useful feature of PCA/NMF is that it allows us to account for the fraction of convoluting signals such as that from the carbon/binder matrix or spectral artifacts when calculating the relative ratio of the LMR-NMC components (i.e. the oxygen oxidation state), which would otherwise require separately isolating and measuring these convoluting signals. We include these extraneous end members to improve the linear combination fit but ignore them when calculating the overall phase fractions of the LMR-NMC components. The quality of the end members identified and the subsequent linear combination fit are evaluated by spatially mapping the fit residual to see if any regions are poorly fit. Supplementary Fig. 17a shows the residuals for the 3 oxygen spectral maps shown in Figure 4c the main text, and shows that the residuals are only slightly increased near the surfaces where the absorption signal is lowest. There are also no significant domains that exhibit high residual. This confirms that an appropriate number of end-members has been selected and that there are no spectral components in the data that are systematically misfit. Supplementary Fig. 17b shows that most pixels have a residual of less than 0.1, which is less than 10% of the 2-norm of the LMR-NMC end-members. Most of the pixels with a larger residual have a brightness below 0.25 (barely visible to the naked eye) and so do not significantly affect the interpretation of the spectral maps.

Once the fractions of the two LMR-NMC components are calculated at each pixel, a color on the HSV color scale is assigned corresponding to the fraction of the reduced phase, such that 0% has a hue of 0 (red) and 100% has a hue of 1/3 (green). Saturation is set uniformly to 100%, and the brightness of each pixel is set to be linearly proportional to the normalization factor – i.e. the integrated area in the 535 – 560 eV (O), 851 – 857 eV (Ni), 776 – 784 eV (Co), or 638 – 647 eV (Mn) range.

Summaries of similar analyses at the TM L edges are shown in Supplementary Fig. 18. The pixel sizes for the Co and Mn data were generally larger than for the Ni and O data and thus the total number of pixels is smaller. This results in a larger fraction of the variance being made up of noise, explaining the smaller values in the Co and Mn cumulative variance plots for a given number of components. In all cases, we attempted NMF with additional components beyond what was suggested by the cumulative variance plots, to confirm that the maximum number of meaningful end-members was identified. Only at the Mn L edge did this result in identifying an additional extreme point and associated end-member. When only two components are used, the second component is a combination of the $Mn^{3+/4+}$ (end-member 2) and saturated Mn (end-member 3) spectra. We attribute this to the fact that the thicker particles exhibited saturation at the Mn edge due to the high Mn content of the LMR-NMC used in this study. Since the effect of saturation is to broaden the Mn L_3 edge spectrum and is similar to the effect of reduction of Mn^{4+} to Mn^{3+}, and since the Mn reduction only occurs in a few surface pixels, from a statistical standpoint the marginal benefit of including a third component to differentiate saturation and Mn reduction is small. It is, however, important for the analysis of the Mn oxidation state, and is therefore included here. As mentioned above, the Mn oxidation state used for the colormaps is defined as the ratio of the Mn^{4+} and $Mn^{3+/4+}$ end-member spectra obtained after performing the linear least squares fit of all three end-members. Thus, in principle, PCA/NMF can calculate the oxidation state of saturated pixels. Nonetheless, the maps shown in Figure 4a are cropped to contain mostly particles that exhibited minimal saturation, thereby avoiding over-interpreting pixels that are dominated by saturation effects and are therefore likely to exhibit substantial error in the calculated oxidation state.

RIXS Map Generation

The data for generating the RIXS maps were collected in the following steps: (1) XAS were collected first on each sample to define the excitation energy range and calibrate the energy values. (2) At each excitation energy with step size of 0.2 eV across the whole O-K near edge regime, the scattered fluorescence photons were collected in the form of 2D image by the Andor CCD detector on the spectrograph. (3) Cosmic ray signals were determined and deducted from the raw 2D images. (4) Intensity of the raw 2D image was directly integrated along the energy channels to generate a single RIXS spectra corresponding to the particular excitation (incident beam) energy. (5) The integrated 1D spectrum was normalized to both the incident beam flux (monitored real-time during the data collection), and the collection time (in seconds). (6) Repeat 2–5 to collect and process the RIXS spectra at all the interested excitation energies, then combine all the processed spectra into a 2D image map, as shown in the figures here. (7) The energy values of the RIXS maps are finally calibrated by following the elastic features (with the same energy on excitation and emission photons) at multiple energies. We note that color scale has been tuned in the figures to emphasize the contrast of intensity. Because the central discussions in this manuscript does not require quantitative analysis of the RIXS line shape, no energy distribution curves (EDCs) were plotted in this paper.

Supplementary References

1. Qiu, B. *et al.* Gas–solid interfacial modification of oxygen activity in layered oxide cathodes for lithium-ion batteries. *Nat. Commun.* **7,** 12108 (2016).
2. Luo, K. *et al.* Charge-compensation in 3d-transition-metal-oxide intercalation cathodes through the generation of localized electron holes on oxygen. *Nat. Chem.* **8,** 684–691 (2016).
3. Luo, K. *et al.* Anion redox chemistry in the cobalt free 3d transition metal oxide intercalation electrode $Li[Li_{0.2}Ni_{0.2}Mn_{0.6}]O_2$. *J. Am. Chem. Soc.* **138,** 11211–11218 (2016).
4. Boulineau, A., Croguennec, L., Delmas, C. & Weill, F. Structure of Li_2MnO_3 with different degrees of defects. *Solid State Ionics* **180,** 1652–1659 (2010).
5. Yabuuchi, N., Yoshii, K., Myung, S.-T., Nakai, I. & Komaba, S. Detailed studies of a high-capacity electrode material for rechargeable batteries, $Li_2MnO_3-LiCo_{1/3}Ni_{1/3}Mn_{1/3}O_2$. *J. Am. Chem. Soc.* **133,** 4404–4419 (2011).
6. Ito, A. *et al.* In situ X-ray absorption spectroscopic study of Li-rich layered cathode material $Li[Ni_{0.17}Li_{0.2}Co_{0.07}Mn_{0.56}]O_2$. *J. Power Sources* **196,** 6828–6834 (2011).
7. Yoon, W.-S. *et al.* Investigation of the charge compensation mechanism on the electrochemically Li-ion deintercalated $Li_{1-x}Co_{1/3}Ni_{1/3}Mn_{1/3}O_2$ electrode system by combination of soft and hard x-ray absorption spectroscopy. *J. Am. Chem. Soc.* **127,** 17479–17487 (2005).
8. Abbate, M. *et al.* Soft-x-ray-absorption studies of the location of extra charges induced by substitution in controlled-valence materials. *Phys. Rev. B* **44,** 5419–5422 (1991).
9. Liu, H. *et al.* Operando lithium dynamics in the Li-rich layered oxide cathode material via neutron diffraction. *Adv. Energy Mater.* **6,** 1502143 (2016).
10. Butorin, S. M., Guo, J., Wassdahl, N. & Nordgren, E. J. Tunable-excitation soft x-ray fluorescence spectroscopy of high-Tc superconductors: an inequivalent-site seeing story. *J. Elec. Spec. Rel. Phenom.* **110-111,** 235–273 (2000).
11. Butorin, S. M. Resonant inelastic x-ray scattering as a probe of optical scale excitations in strongly electron-correlated systems: quasi-localized view. *J. Elec. Spec. Rel. Phenom.* **110-111,** 213–233 (2000).
12. Thackeray, M. M. *et al.* Li_2MnO_3-stabilized $LiMO_2$ (M = Mn, Ni, Co) electrodes for lithium-ion batteries. *J. Mater. Chem.* **17,** 3112 (2007).
13. Shukla, A. K. *et al.* Unravelling structural ambiguities in lithium- and manganese-rich transition metal oxides. *Nat. Commun.* **6,** 8711 (2015).
14. Lee, E. & Persson, K. A. Structural and chemical evolution of the layered Li-excess Li_xMnO_3 as a function of Li content from first-principles calculations. *Adv. Energy Mater.* **4,** 1400498 (2014).
15. Lim, J.-M. *et al.* The origins and mechanism of phase transformation in bulk Li_2MnO_3: first-principles calculations and experimental studies. *J. Mater. Chem. A* **3,** 7066–7076 (2015).
16. Reed, J. & Ceder, G. Role of electronic structure in the susceptibility of metastable transition-metal oxide structures to transformation. *Chem. Rev.* **104,** 4513–4534 (2004).
17. Chen, H. & Islam, M. S. Lithium extraction mechanism in Li-rich Li_2MnO_3 involving oxygen hole formation and dimerization. *Chem. Mater.* **28,** 6656–6663 (2016).
18. Gao, Y., Ma, J., Wang, Z., Lu, G. & Chen, L. Vacancy-induced MnO_6 distortion and its

impacts on structural transition of Li_2MnO_3. *Phys. Chem. Chem. Phys.* **19,** 7025–7031 (2017).
19. Xie, Y., Saubanère, M. & Doublet, M. L. Requirements for reversible extra-capacity in Li-rich layered oxides for Li-ion batteries. *Energy Environ. Sci.* **10,** 266–274 (2017).
20. McCalla, E. *et al.* Visualization of O-O peroxo-like dimers in high-capacity layered oxides for Li-ion batteries. *Science* **350,** 1516–1521 (2015).
21. Hong, J., Gwon, H., Jung, S.-K., Ku, K. & Kang, K. Review—lithium-excess layered cathodes for lithium rechargeable batteries. *J. Electrochem. Soc.* **162,** A2447–A2467 (2015).
22. Ishida, N., Tamura, N., Kitamura, N. & Idemoto, Y. Crystal and electronic structure analysis and thermodynamic stabilities for electrochemically or chemically delithiated $Li_{1.2-x}Mn_{0.54}Ni_{0.13}Co_{0.13}O_2$. *J. Power Sources* **319,** 255–261 (2016).
23. Mohanty, D. *et al.* Neutron diffraction and magnetic susceptibility studies on a high-voltage $Li_{1.2}Mn_{0.55}Ni_{0.15}Co_{0.10}O_2$ lithium ion battery cathode: insight into the crystal structure. *Chem. Mater.* **25,** 4064–4070 (2013).
24. Jolliffe, I. T. *Principal Component Analysis* (Springer-Verlag New York Inc., New York, 2002).
25. Wise, A. M. *et al.* Nanoscale chemical imaging of an individual catalyst particle with soft x-ray ptychography. *ACS Catal.* **6,** 2178–2181 (2016).
